# Heart Failure Care and Post-Hospital Outcomes in Northwestern Tanzania

**DOI:** 10.5334/gh.1593

**Published:** 2026-10-26

**Authors:** Fredrick Kalokola, Bahati K. Wajanga, Mabula Deodatus, Eva Mujuni, Patrick Mayanja, David Majule, Andrew Luhanga, Evarist B. Msaki, Audax Malulu, Mariam Goba, Fortunatus Nestory, Jimmyson Masinga, Larry A. Allen, Priya B. Shete, Jeffrey Olgin, Robert Peck, Cody Cichowitz

**Affiliations:** 1 Department of Medicine, Bugando Medical Centre, Mwanza, Tanzania; 2 Division of Cardiology, Department of Medicine, University of Colorado School of Medicine, CU Anschutz, Aurora, Colorado, United States; 3 Division of Pulmonary and Critical Care Medicine, San Francisco General Hospital, University of California San Francisco, San Francisco, United States; 4 Division of Cardiology, University of California San Francisco, San Francisco, United States; 5 Center for Global Health, Weill Cornell Medicine, New York, United States; 6 Center for Global Health, Colorado School of Public Health, Aurora, Colorado, United States

**Keywords:** heart failure, global health, Africa, post-hospital outcomes, implementation science

## Abstract

**Introduction:**

In Africa, heart failure (HF) mortality is the highest in the world; approximately 50% of patients die within one year of HF hospitalization. Little is known about the quality of inpatient and post-discharge care and its association with patient outcomes. The purpose of this study is to describe HF outcomes for patients admitted in Northwestern Tanzania and evaluate associations between gaps in quality care and post-hospital survival.

**Methods:**

We conducted a retrospective chart review of all adult patients admitted with HF at Bugando Medical Centre in 2023. We abstracted data from the index hospitalization and subsequent encounters until August 31, 2025. Vital status was ascertained by telephone. We characterized post-discharge outcomes and adherence to guideline recommendations for HF care. Cox proportional hazards models assessed associations between gaps in quality measures and mortality.

**Results:**

Among 177 patients admitted with HF, 23 (13%) died during the index hospitalization. Of the 154 patients alive at discharge, 63 (40.9%) died within six months and 86 (56%) within a year. Among patients alive at discharge, 79% received indicated testing, 50% were on guideline-directed medical therapy (GDMT) at discharge, 68% attended the first post-discharge appointment, 53% received ongoing care three months post-discharge, and 39% were on GDMT three months post-discharge. Attending the first post-discharge visit (aHR 0.32 [95% CI: 0.17, 0.63]) and remaining in care three months after discharge (aHR 0.36 [95% CI: 0.16, 0.81]) were both independently associated with improved survival. GDMT use was low across outpatient visits (47%), with frequent medication changes and de-escalation over time.

**Conclusions:**

Mortality following HF admission in Northwestern Tanzania remains high. Factors associated with post-hospital mortality may include poor linkage to post-discharge care and low sustained care engagement. Implementation research is needed to develop strategies to improve post-discharge HF outcomes in East Africa, focusing on optimizing GDMT before discharge and improving early post-hospital linkage to care.

**Lay Summary:**

- Heart failure (HF) mortality in sub-Saharan Africa is the highest in the world, yet little is known about the quality of HF care provided during and after HF hospitalization and its relationship to post-hospital outcomes. This study seeks to understand the quality of care provided to patients admitted with HF in Northwestern Tanzania and its association with post-hospital outcomes.
- This study took place at Bugando Medical Centre, a large academic regional referral hospital in Mwanza, Tanzania that serves a catchment population of ~22 million people. Among 177 patients admitted with HF in 2023, there was a trend of high, early mortality following hospital discharge. Among the 154 patients who survived and were discharged, we found significant gaps in linkage to care after hospitalization, sustained retention in care, and utilization of guideline-directed medical therapy at discharge and in the outpatient setting. Linkage to care and retention in care were each independently associated with significant improvements in survival.
- There is a pressing need for future studies focused on understanding and addressing factors associated with post-hospital care engagement and utilization of guideline-directed medical therapy after HF hospitalization in East Africa.

## Graphic Abstract

**Figure:**
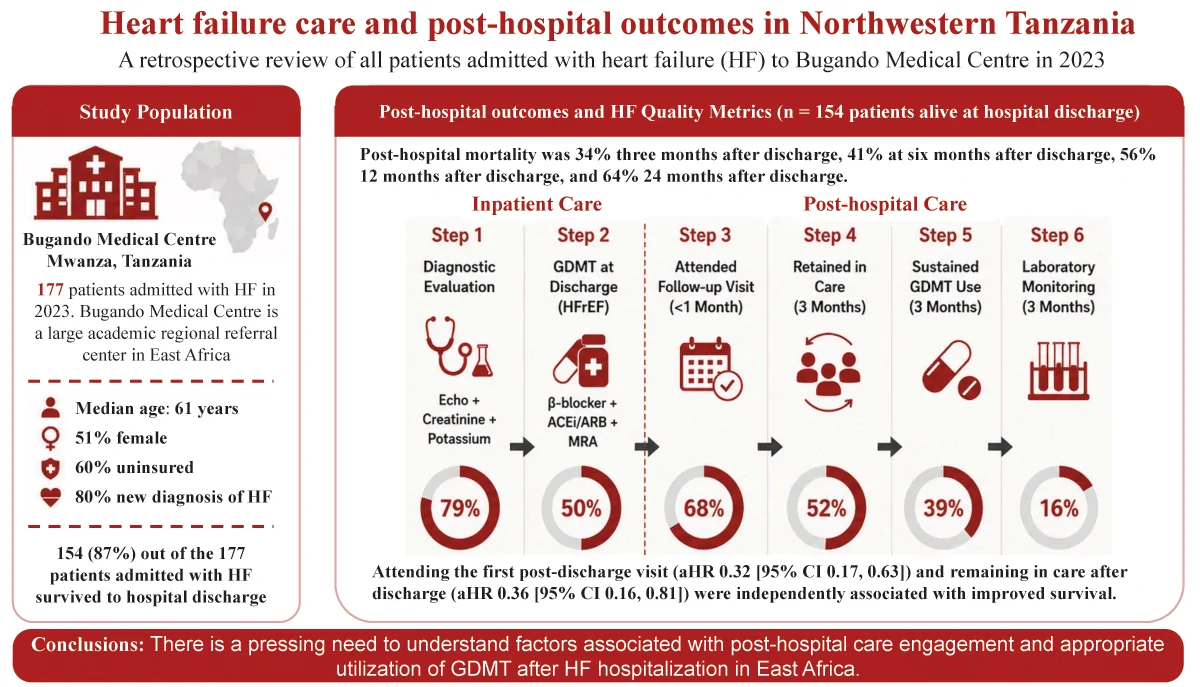


## Introduction

Globally, the prevalence of heart failure (HF) continues to increase. HF currently affects approximately 1–3% of adults and accounts for an annual economic burden of ~284 billion USD (1,2,3). Improvements have been made in the diagnosis and treatment of HF; however, large disparities in access to quality care and subsequent outcomes persist (1,4). Data from two global HF cohorts—the Global Congestive Heart Failure registry and International Congestive Heart Failure cohort—highlighted that life-saving guideline-directed medical therapy (GDMT) is used less frequently in low- and middle-income countries (LMICs) compared to high-income countries (5,6), and that HF patients in LMICs suffer significantly higher mortality— approximately 2.5 times what is observed in high-income countries (5). In sub-Saharan Africa, up to 50% of patients admitted with acute HF die within a year of diagnosis, and HF-related mortality and age-standardized years lived with disability (YLDs) are the highest in the world (3,4).

To address these disparities and improve care for HF and other non-communicable diseases, the World Health Organization (WHO) released the Package of Essential Noncommunicable (PEN) disease interventions in 2020 as a strategy for countries to improve implementation of non-communicable disease care (7). This was further expanded by the Non-Communicable Disease and Injury Poverty Network PEN-plus program (8), which was adopted by all member states in the WHO African Region in 2022 and stipulates that HF care be made available at all hospitals (9). The guidelines stipulate that patients with suspected HF should receive an echocardiogram (echo), renal function testing (measurement of serum creatinine and potassium), and be initiated on standard GDMT, which, for patients with reduced left ventricular ejection fraction, includes beta-blockers, ACE inhibitors (ACEi) or angiotensin receptor blockers (ARBs), and mineralocorticoid receptor antagonists (MRAs).

Since the adoption of PEN-plus, there are little to no data to describe gaps in implementation. In Africa, data on HF care has largely focused on inpatient and short-term survival (10). There is a pressing need to identify gaps in the implementation of guideline-recommended HF care and to identify which gaps are most strongly associated with morbidity and mortality for people with HF. The purpose of this retrospective cohort study is to describe HF care and outcomes for patients admitted at an academic, regional referral hospital in Northwestern Tanzania and characterize how gaps in the implementation of HF guidelines relate to post-hospital mortality.

## Methods

### Study setting and population

We conducted a retrospective analysis of HF patients admitted to the adult medical ward at Bugando Medical Centre (BMC) between January 1 and December 31, 2023. BMC is an academic quaternary care center in the city of Mwanza. It serves as the referral center for eight regions across the Lake Zone of Tanzania, with a population of approximately 22 million people. Patients were eligible for inclusion in this study if they were diagnosed with HF at any point during their admission to the adult medical ward; there were no exclusion criteria. A diagnosis was made by the admitting or treating clinician; per standard clinical practice in Tanzania, this diagnosis was based upon the Framingham criteria (11).

The study was reviewed by the institutional review board at the Catholic University of Allied Health Sciences Bugando and the Weill Cornell School of Medicine. Since this is a retrospective review of de-identified existing clinical data, informed consent was not required from participants.

### Study procedures

At the time of HF diagnosis during admission to the adult medical ward (patients aged >13), a physician and member of the cardiology care team captured the patients’ medical record numbers and up to three separate telephone numbers per standard protocol at BMC. Telephone numbers were for the patient and their designated contact(s).

During a one-month period (September 2025), data were abstracted from the electronic medical record by a cardiologist and member of the study team (Cody Cichowitz) using a data abstraction tool programmed into REDCap. Data were abstracted from the time of the index admission in 2023 and all subsequent care encounters occurring up until August 31, 2025.

### Data abstraction

Data abstracted from the index admission included age, sex, health insurance status, the results of diagnostic testing, vital signs, and discharge medications. All notes from the index hospitalization were reviewed together with the echo report. Participants were categorized as having HF with reduced ejection fraction (HFrEF: left ventricular ejection fraction <40%), HF with mildly reduced ejection fraction (HFmrEF: left ventricular ejection fraction between 41–49%), and HF with preserved ejection fraction (HFpEF: left ventricular ejection fraction >50%). Available laboratory data included the hemoglobin at the time of admission and all creatinine and potassium levels measured during hospitalization. Comorbid chronic conditions captured included hypertension, diabetes, atrial fibrillation or flutter, and HIV. The diagnosis of comorbid conditions was made by the treating physician at the index hospitalization and abstracted from the electronic medical record using notes and ICD codes. Data abstracted from post-discharge encounters included vital signs, basic laboratory data, HF prescriptions, any subsequent readmissions, and in-hospital deaths.

### Heart failure care metrics

We characterized the proportion of patients who received quality care as stipulated by current HF guidelines (7,8,9). We then enumerated the proportion of patients who received appropriate diagnostic testing with an echo and measurement of serum creatinine and potassium; were diagnosed with HFrEF and prescribed GDMT prior to discharge; attended post-discharge follow-up within one month of discharge; were retained in outpatient care three months after discharge; received GDMT for the first three months after discharge (HFrEF patients only); and had repeat measurement of creatinine and potassium within the first three months following discharge and GDMT initiation.

Within each class of medication used in GDMT, appropriate use was defined as the medication being prescribed and filled by the pharmacy or there being a contraindication to using that class of medication as described in the PEN-plus guidelines (7,8,9). For example, a patient with HFrEF was considered to have appropriate use of beta-blockers if they were either (a) prescribed or (b) not prescribed, and the heart rate was less than 55 beats per minute. Similarly, ACEi and ARBs are contraindicated in the PEN-plus guidelines if serum creatinine is greater than 2.3 mg/dL or the systolic blood pressure is less than 100 mmHg, while MRAs are contraindicated if the serum creatinine is greater than 2.3 mg/dL or the serum potassium is greater than 5.5 mEq/L. Additionally, sodium-glucose co-transporter 2 inhibitors were not widely available in Tanzania in 2023, nor covered by insurance. Thus, GDMT in this study was defined to be combination therapy with beta-blockers, ACEi or ARBs, and MRAs. Assessment of GDMT would ideally include evaluation of titration, but we observed that very few participants were prescribed GDMT consistently. Therefore, we did not include medication titration in this analysis.

### Post-discharge events

Following medical record abstraction, all patients in the registry were contacted by a physician and member of the cardiology care team at BMC (Mariam Goba) using the phone numbers provided during their index admission, along with any additional numbers documented in their medical record. To maximize response rates, each participant was called on three separate days at different times of day. If a participant or their family member was reachable, vital status was recorded, along with any known hospital readmissions, ongoing care engagement for HF, and medication use.

For the subset of patients who could not be reached by phone, two additional steps were taken: For patients with health insurance, we contacted the health insurance company to ascertain vital status. For patients who were not reachable and did not have health insurance, two members of the study team independently reviewed all available electronic medical records—including severity of heart failure, comorbid conditions, vital signs, and medication prescription data—and categorized vital status as ‘likely dead’ or ‘unknown’; any disagreements were adjudicated by a third reviewer. Because phone numbers in Tanzania only remain active if they are used every three months, and each number is connected to mobile banking, it is unlikely that patients would lose access to all mobile numbers if they were still alive.

### Data analysis

Standard descriptive statistics were used to characterize patients admitted with HF at BMC during 2023. We described inpatient and post-hospital mortality and evaluated factors associated with death during the index hospital HF hospitalization or after discharge. Univariate and multivariable logistic regressions were performed to assess for factors associated with inpatient mortality. Cox proportional hazards regression analyses were performed to assess for factors associated with post-hospital mortality. Exposures of interest were defined *a priori* and included age, sex, insurance status, timing of HF diagnosis (i.e., established or new diagnosis), HFrEF, anemia, chronic kidney disease, hypertension, diabetes, and atrial fibrillation or flutter. The proportional hazards assumption was assessed using the Grambsch–Therneau test based on scaled Schoenfeld residuals, evaluated both globally and for each individual covariate, and supplemented by visual inspection of scaled Schoenfeld residual plots against time and log-minus-log survival curves. For models exhibiting non-proportionality, sensitivity analyses incorporating time-varying covariates were performed to assess the temporal stability of the hazard ratios. Complete data were available for each of these exposures except for ejection fraction (11.9% unavailable), kidney function (7.3% unavailable), and hemoglobin (5.6% unavailable); data were unavailable because these diagnostic tests were not completed. In the primary analyses, missing data were assumed to be normal. We performed two secondary analyses: one restricted to participants with complete data and the other wherein missing data were assumed to be abnormal.

In the primary analysis of post-hospital mortality, participants who were not reachable over the phone were censored at their last known care encounter. We conducted two pre-specified sensitivity analyses. In the first, patients were considered dead if (a) death was confirmed during data collection or (b) they were out of care, not reachable, and their vital status was determined to be ‘likely dead’ during vital status adjudication by the study team. In the second sensitivity analysis, we assumed that all participants who were out of care and not reachable were dead.

We next assessed whether each discrete step in HF care was associated with post-hospital mortality using univariate and multivariate Cox proportional hazards regression analyses, adjusting for the same variables included in the base model evaluating for factors associated with post-hospital outcomes. In the regressions that assessed for an association between attending the first post-discharge appointment and retention in care at three months and survival, we restricted the analyses to only include people who survived at least one month and three months after hospital discharge, respectively. GDMT utilization was further characterized at the time of hospital discharge, any subsequent readmissions, and from all outpatient clinic visits. All analyses were completed in STATA 19.

## Results

### Study population

Between January 1 and December 31, 2023, a total of 177 patients were admitted to BMC with a primary diagnosis of HF (Table 1). The median age was 61.0 (IQR: 45.9, 72.8) years. Women comprised half of the cohort (50.8%). Among the patients, 59.9% were without health insurance/self-paying, and 79.7% were diagnosed with HF for the first time during index hospitalization. Approximately half of patients (48.6%) had HFrEF, 12.4% had HFmrEF, and 27.1% had HFpEF; 11.9% of patients admitted with HF never received an echo. Hypertension (46.9%) and chronic kidney disease (CKD) at Stage 3 or above (65.0%) were the most common comorbidities, followed by diabetes (18.1%) and atrial fibrillation or flutter (17.5%).

**Table 1:** Patients admitted to BMC with heart failure during 2023 (n = 177).


	OVERALL (n = 177)	SURVIVED INDEX ADMISSION (n = 154)	DIED DURING INDEX ADMISSION (n = 23)	P-value*

**Age (years)**	61.0 (45.9, 72.8)	61.6 (47.7, 72.1)	58.0 (25.0, 76.0)	0.37

**Female [ref: male]**	90 (50.8%)	83 (53.9%)	7 (30.4%)	0.04

**Self-pay [ref: insured]**	106 (59.9%)	92 (59.7%)	14 (60.9%)	0.92

**New heart failure diagnosis** **[ref: established diagnosis]**	141 (79.7%)	126 (81.8%)	15 (65.2%)	0.089

**LV ejection fraction (%)**	40 (29.0, 55.0)	40.0 (29.0, 55.0)	35.0 (29.0, 59.5)	0.80

No echo completed	21 (11.9%)	13 (8.4%)	8 (34.8%)	0.70

HFpEF: LV EF *>* 50%	48 (27.1%)	44 (28.6%)	4 (17.4%)	

HFmrEF: LV EF 41–49%	22 (12.4%)	21 (13.6%)	1 (4.3%)	

HFrEF: LV EF *<* 40%	86 (48.6%)	76 (49.4%)	10 (43.5%)	

**Comorbidities**				

HTN	83 (46.9%)	77 (50.0%)	6 (26.1%)	0.04

Diabetes	32 (18.1%)	30 (19.5%)	2 (8.7%)	0.26

Atrial fibrillation or flutter	31 (17.5%)	24 (15.6%)	7 (30.4%)	0.14

HIV	6 (3.4%)	4 (2.6%)	2 (8.7%)	0.18

**Laboratory data on admission**				

Kidney function: eGFR (mL/min/1.73 m²)	51.8 (31.6, 74.0)	53.4 (34.3, 76.4)	45.4 (20.3, 55.9)	0.06

eGFR not measured	13 (7.3%)	11 (7.1%)	2 (8.7%)	0.06

CKD Stage 1 and 2 (eGFR *>* 60 mL/min/1.73 m²)	62 (35.0%)	58 (37.7%)	4 (17.4%)	

CKD Stage 3 (eGFR 30–59 mL/min/1.73 m²)	62 (35.0%)	54 (35.1%)	8 (34.8%)	

CKD Stage 4 (eGFR 15–29 mL/min/1.73 m²)	24 (13.6%)	20 (13.0%)	4 (17.4%)	

CKD Stage 5 (eGFR <15 mL/min/1.73 m²)	16 (9.0%)	11 (7.1%)	5 (21.7%)	

Hemoglobin (mg/dL)	12.1 (10.4, 13.5)	12.1 (10.5, 13.4)	12.3 (10.2, 13.8)	0.97

Hb not measured	10 (5.6%)	9 (5.8%)	1 (4.3%)	0.58

Normal or mild anemia: (Hb *>* 11 mg/dL)	115 (65.0%)	101 (65.6%)	14 (60.9%)	

Moderate anemia: (Hb: 8–10.9 mg/dL)	44 (24.9%)	38 (24.7%)	6 (26.1%)	

Severe anemia: (Hb < 8.0 mg/dL)	8 (4.5%)	6 (3.9%)	2 (8.7%)	


*P-values derived from Fisher’s exact test for categorical variables and Wilcoxon rank-sum for continuous variables.

### Heart failure outcomes

Of the 177 patients who were admitted with HF, 23 (12.9%) died during the index admission, and 154 (87.1%) survived to discharge (Table 1). Among the 154 patients who survived to be discharged, vital status was ascertained for 110 patients by phone call or hospital/insurance records review. For the remaining 44 participants, the study team adjudicated outcomes as ‘likely dead’ for 37 patients and ‘unknown’ for 7 patients. There were high rates of early mortality (Figure 1); 24 months after discharge, 63.6% of patients were dead (98 out of 154; 61 confirmed deaths and 37 likely deaths).

**Figure 1:**
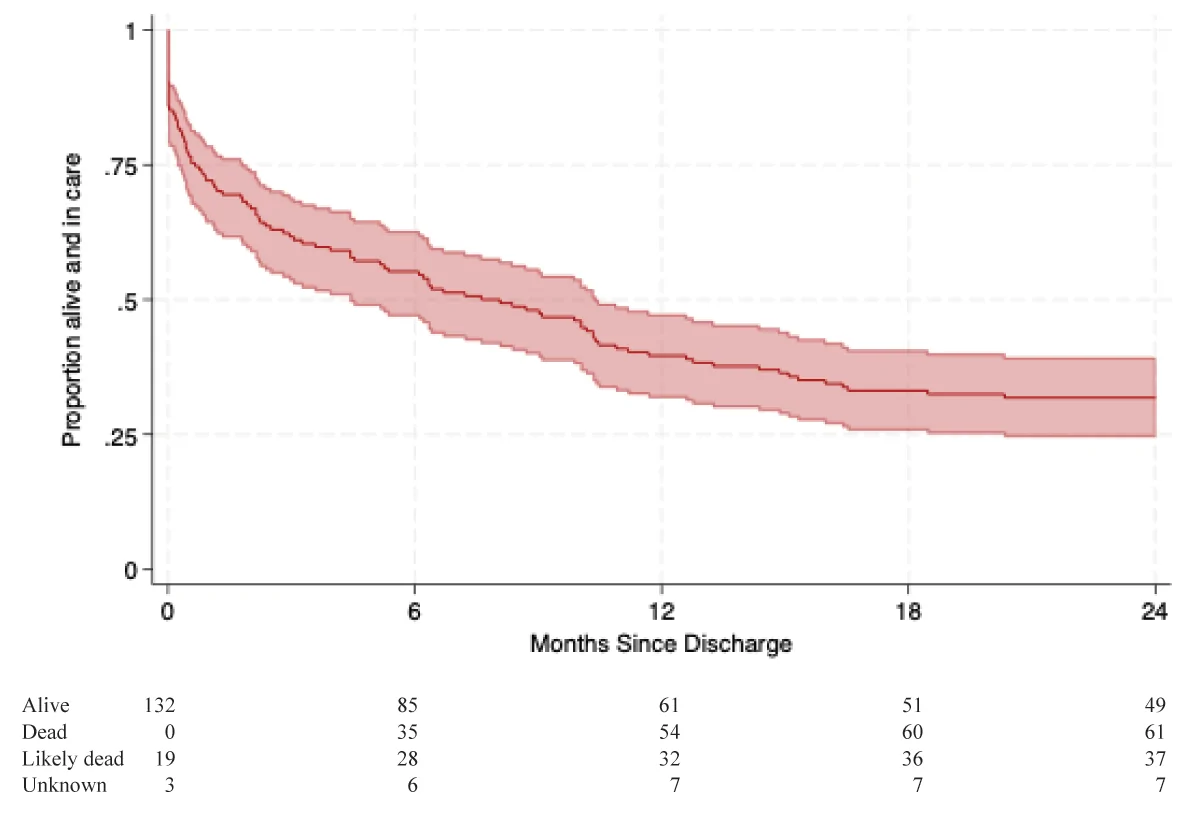
Post-hospital survival for patients alive at the time of discharge from index admission (n = 154). Kaplan-Meier curve demonstrating the proportion of patients alive and in care following discharge from the index hospitalization. For patients who were not reachable by phone and out of care for at least 3 months, likely vital status was determined by two members of the study team who independently reviewed all available data and categorized as “likely dead” or “unknown”. Disagreements were adjudicated by a third reviewer.

The presence of renal dysfunction on admission was the only exposure independently associated with inpatient mortality (Supplemental Table 1). Specifically, compared to patients with CKD Stage 1 or 2, patients with CKD Stage 5 (aOR 8.62 [95% CI: 1.82, 40.73]) had a significantly increased risk of death. A similar trend was seen for patients with CKD Stage 3 (aOR 3.80 [95% CI: 0.97, 14.81]) and 4 (aOR 4.28 [95% CI: 0.78, 23.48]), but this association was not significant. Age, ejection fraction, insurance status, and the presence of comorbid hypertension, diabetes, or atrial fibrillation were not associated with an increased risk of inpatient mortality.

In adjusted Cox regressions (Supplemental Table 1), patients with CKD Stage 5 had an increased hazard of death during the post-hospital period (aHR 2.56 [95% CI: 1.09, 6.00]). There were no statistically significant differences in the risk of death for patients based upon insurance status (Figure 2-Panel A), those with an LVEF <40% (Figure 2-Panel B), CKD, anemia or comorbid hypertension, diabetes, or atrial fibrillation. In sensitivity analyses where different outcomes were considered for participants who were out of care and not reachable by phone (Supplemental Table 2; Model 2 and Model 3), there were no qualitative differences in the findings. Similarly, in sensitivity analyses where participants with missing data were removed from the analysis (Supplemental Table 3) or where missing data were assumed to be abnormal (Supplemental Table 4), there were no qualitative differences in the findings. In Figure 2-Panel D, we display post-discharge outcomes compared to data from a prior study that examined post-hospital mortality among HF patients at BMC in 2013 (12). Over the 10 years separating the two cohorts, there was no improvement in post-discharge survival (log-rank p-value 0.33).

**Figure 2:**
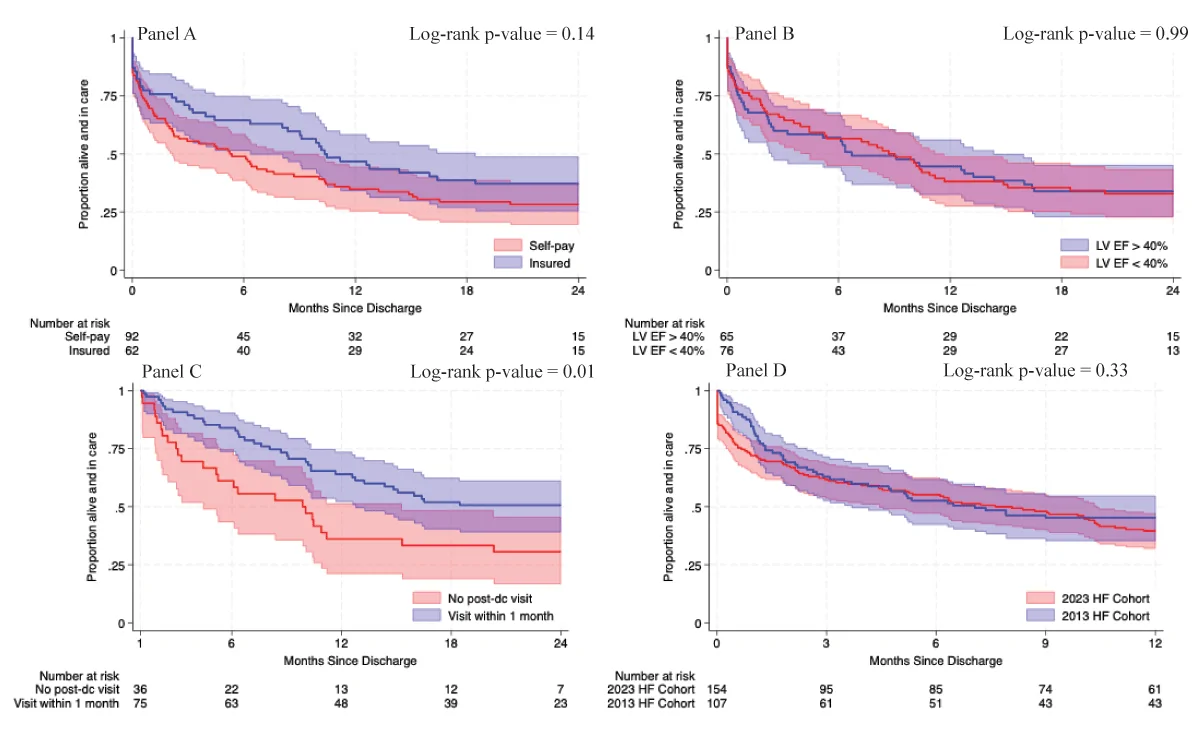
Post-hospital survival by key exposures. Time to all cause mortality after hospital discharge by key exposures including: insurance status **(Panel A)**, ejection fraction **(Panel B)**, and follow up within the first month after discharge among the 111 patients who survived at least one month after the index hospitalization **(Panel C)**. Of note, every patient is scheduled for post-discharge (post-dc) follow up within 2 weeks of hospital discharge. In **Panel D**, we compare the data from the 2023 HF Cohort to data from a historical HF Cohort that collected data in 2013.

### Gaps in quality heart failure care and their association with post-hospital survival

We identified significant gaps at each step of guideline-directed HF care for patients who survived to discharge (Figure 3), of which post-discharge follow-up and retention in care three months after discharge were associated with improvements in post-hospital survival (Table 2). In Step 1, 79.2% of patients admitted with HF underwent the minimal recommended testing prior to discharge. Receiving recommended testing prior to discharge was not associated with a change in post-hospital survival (aHR 1.27 [95% CI: 0.66, 2.41]). In Step 2, 50% of patients with HFrEF were on appropriate GDMT at the time of discharge; this was not associated with a change in post-discharge survival (aHR 0.98 [95% CI: 0.42, 2.30]).

**Figure 3:**
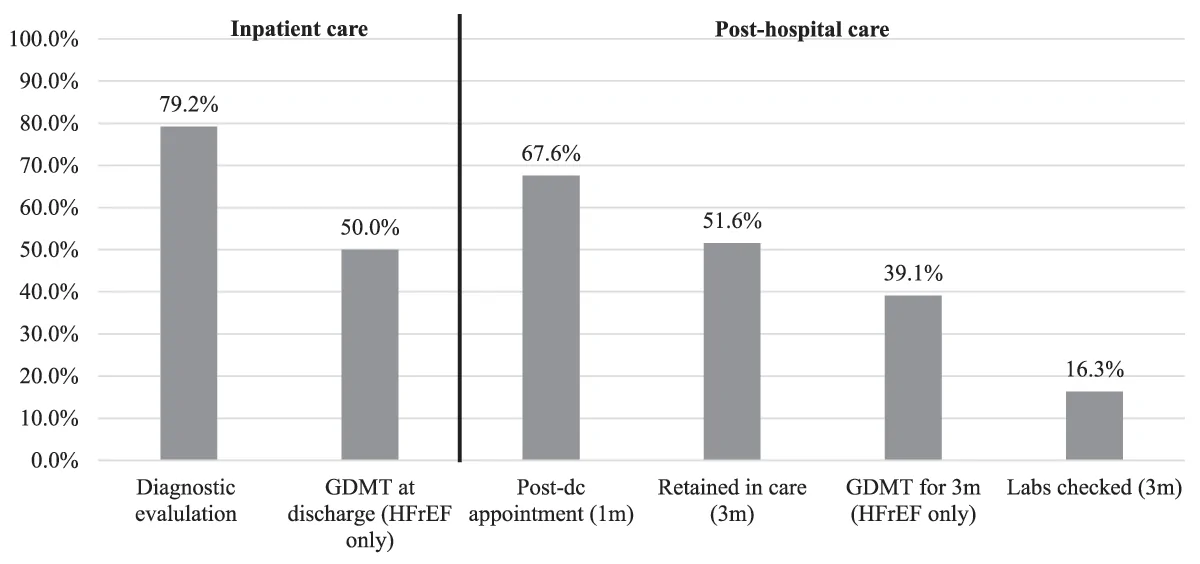
Gaps in delivery of quality heart failure care. This figure displays the proportion of patients that received each step of quality heart failure care described by PEN-plus guidelines. **Step 1:** proportion of patients who underwent guideline-directed testing, including measurement of serum creatinine and potassium and an echo during or prior to hospitalization (out of 154 patients who survived to discharge). **Step 2:** proportion of HFrEF patients prescribed appropriate GDMT at time of discharge (out of 76 HFrEF patients who survived to discharge). **Step 3:** proportion of patients who attended their scheduled outpatient visit within the first month after discharge (out of the 111 patients who survived at least a month after discharge). **Step 4:** proportion of patients who were retained in care for 3 months after discharge (out of the 95 patients who survived at least 3 months after discharge). **Step 5:** proportion of HFrEF patients on appropriate GDMT at each clinic visit in the first three months after discharge (out of 23 HFrEF patients retained in care at 3 months). **Step 6:** proportion of patients, who had labs checked (out of the 49 patients who were retained in care at 3 months).

**Table 2:** **Gaps in heart failure care and post-hospital survival**. The adjusted hazard ratios presented were derived by running a multivariable Cox regression model for each element of the heart failure care continuum, adjusted for age, sex, insurance status, timing of heart failure diagnosis, HFrEF, hypertension, diabetes, atrial fibrillation or flutter, CKD, and anemia. These same variables were used in the adjusted base models shown in Supplemental Table 1. The adjusted model for Step 1 was not adjusted for HFrEF, CKD, and anemia, as these were collinear with the exposure of interest. Participants who could not be reached by phone and were out of care for at least 3 months were handled as follows in each model:


	MODEL 1		MODEL 2	MODEL 3
		
	UNADJUSTED HR	ADJUSTED HR [95% CI]	ADJUSTED HR [95% CI]	ADJUSTED HR [95% CI]

**Step 1:** Diagnostic evaluation before discharge (n = 154 patients who survived to discharge)	1.19 [0.64, 2.23]	1.27 [0.66, 2.41]*	1.08 [0.63, 1.87]*	1.12 [0.65, 1.94]*

**Step 2:** GDMT at discharge (n = 76 HFrEF patients who survived to discharge)	1.17 [0.58, 2.34]	0.98 [0.42, 2.30]	1.00 [0.48, 2.10]	1.06 [0.51, 2.21]

**Step 3:** Attended post-discharge appointment within one month (n = 111 patients who survived at least one month)	0.42 [0.24, 0.73]	0.32 [0.17, 0.63]*	0.50 [0.29, 0.89]	0.51 [0.29, 0.90]

**Step 4:** Retained in care for the first three months after discharge (n = 95 patients who survived at least three months)	0.43 [0.22, 0.85]	0.36 [0.16, 0.81]	0.50 [0.26, 0.98]	0.49 [0.25, 0.96]

**Step 5 and 6:** We did not evaluate the association of GDMT for three months or repeating labs within the first three months of discharge (e.g., safety monitoring) and survival due to the limited sample size.				


Model 1: Censored at the time of their last contact with the health care system. Model 2: Vital status adjudicated by the study team. Model 3: Assumed to be dead. *In all but these four models, the Grambsch-Therneau test results supported the proportionality assumption (p > 0.05). For the remaining four models, the global test indicated evidence of non-proportionality driven primarily by the primary exposure (p < 0.05), but residual plots demonstrated that the log-minus-log curves did not cross one another, indicating that departures from proportionality were modest. For these models, the reported hazards ratio is interpreted as a weighted average of the time-varying hazards ratio over follow-up, rather than a constant effect. Models incorporating time-varying exposure interaction yielded consistent qualitative findings; primary proportional hazards ratio models are presented for clarity and parsimony.

In Step 3, 67.6% of those who survived at least one month after discharge attended their scheduled post-hospital follow-up appointment. In an adjusted Cox regression analysis restricted to patients who survived at least one month after discharge, those who attended their appointment had a substantial improvement in survival between 30 days and 31 months after discharge (aHR 0.32 [95% CI: 0.17, 0.63]; Figure 2-Panel C). In Step 4, 51.6% of those who survived three months after discharge were retained in care and had attended every scheduled outpatient appointment. Retention in care for the first three months after discharge was also associated with a significant improvement in mortality between 90 days and 31 months after discharge (aHR 0.36 [95% CI 0.16, 0.81]). In Step 5, 39.1% of HFrEF patients retained in care for three months after discharge were on GDMT. Finally, in Step 6, 16.3% of patients retained in care for three months had repeat measurement of serum creatinine and potassium after hospital discharge. The survival benefit associated with attending an early post-discharge follow-up appointment and remaining in care for three months was consistent in pre-defined sensitivity analyses (Table 2) that considered different possible outcomes for patients who were out of care and not reachable by phone, highlighting the strength and consistency of this finding across models.

### Utilization of GDMT

Among the 154 patients who survived to hospital discharge, 76 (49.5%) had HFrEF. During the study period, these 76 patients discharged with HFrEF had 60 readmissions and 453 clinic visits. The utilization of GDMT for patients with HFrEF at both hospital discharge and outpatient clinic visits was low (Figure 4), with the largest gaps observed in the use of beta-blockers and MRAs. Overall, patients were more likely to be prescribed all three classes of GDMT at outpatient clinic appointments (46.6% of all visits; 211 out of 453; Figure 4b) than at hospital discharge (28.7% of all discharges; 39 out of 136; Figure 4a). Importantly, and unexpectedly, self-pay patients were slightly more likely to receive GDMT than patients with insurance, with one notable exception: insured patients received beta-blockers in outpatient clinics (79% of visits) much more frequently than self-pay patients (51% of visits). Overall, the proportion of patients on all three classes of evidence-based therapies was low.

**Figure 4a:**
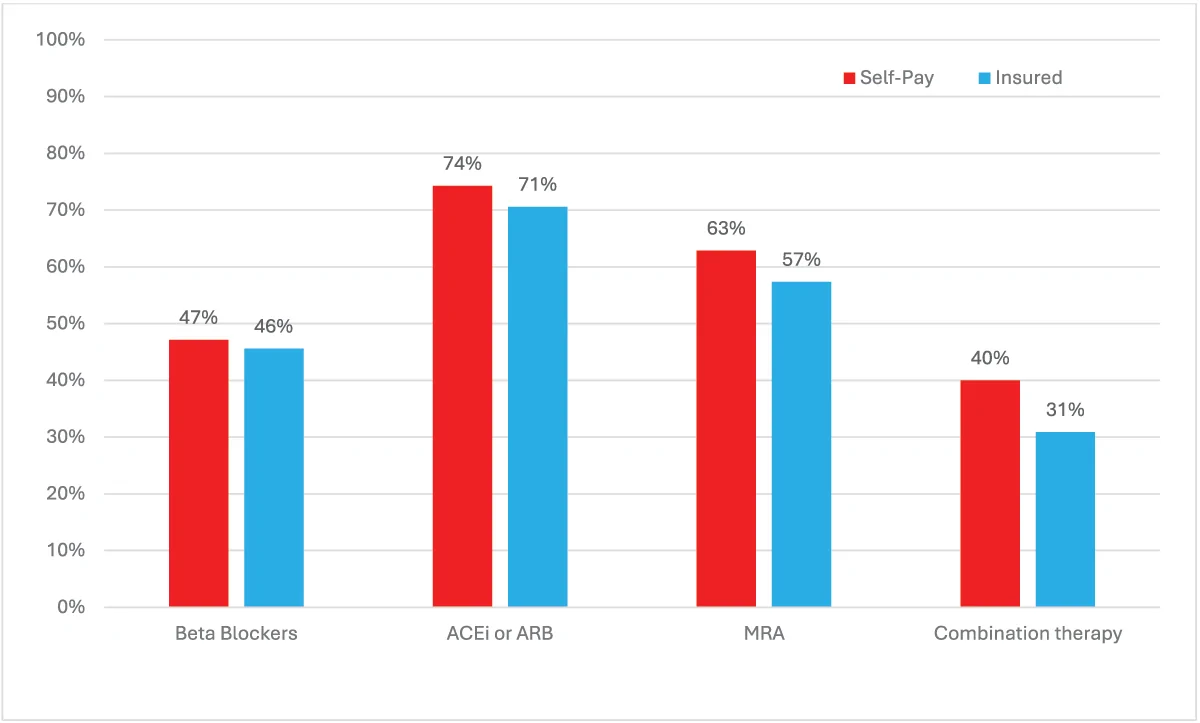
GDMT use for 76 patients with HFrEF at Hospital Discharge (n = 136 discharges*).

**Figure 4b:**
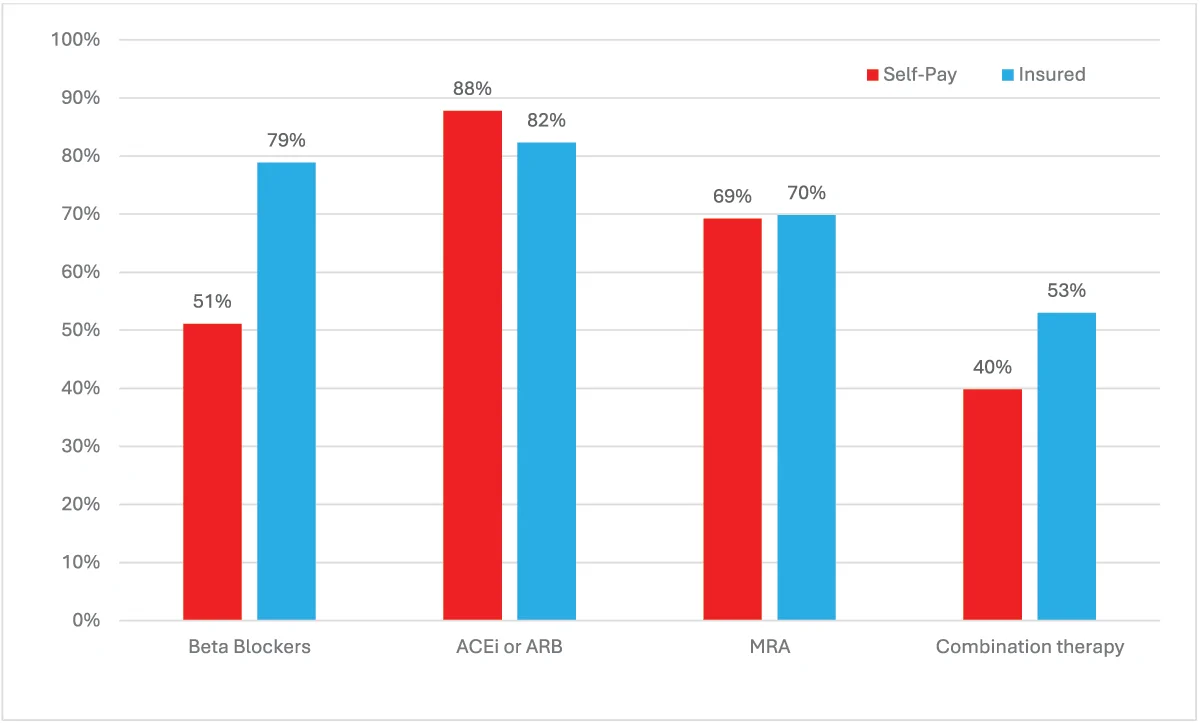
GDMT use for 76 patients with HFrEF at Outpatient Clinic Visits (n = 453 clinic visits). Figure 4 describes GDMT use across all discharges and outpatient clinic visits for 76 participants with HFrEF who survived to hospital discharge (33 insured patients; 43 self-pay patients). Each class of medication was considered appropriately used if it was dispensed at the pharmacy or if there was a documented contraindication to a particular class of medications as detailed in the PEN-Plus heart failure guidelines (e.g. bradycardia for beta blockers or severe renal dysfunction for ACEi or ARBs). *Data from 136 discharges derived from 76 discharges from the index admission and 60 heart failure related readmissions.

Figure 5 demonstrates patterns of GDMT utilization among the 76 patients with HFrEF who survived to discharge. Each row in Figure 5 represents an individual patient, and each column represents a sequential month after index hospitalization. In the first column, which represents discharge from the index hospitalization, only 50% of patients were on all three classes of recommended GDMT; however, almost all the patients were on at least one HF medication. Similar to what was observed in Table 2 and Figure 2-Panel C, patients at the bottom of the heat map who had better care engagement were most likely to survive, even though month-to-month changes in GDMT occurred frequently, and most patients were often on fewer classes of medications as time went on. No patients received all three classes of GDMT (green in each column) throughout the duration of follow-up. Patients at the top of the figure, who died during follow-up, generally had little to no post-hospital care engagement.

**Figure 5:**
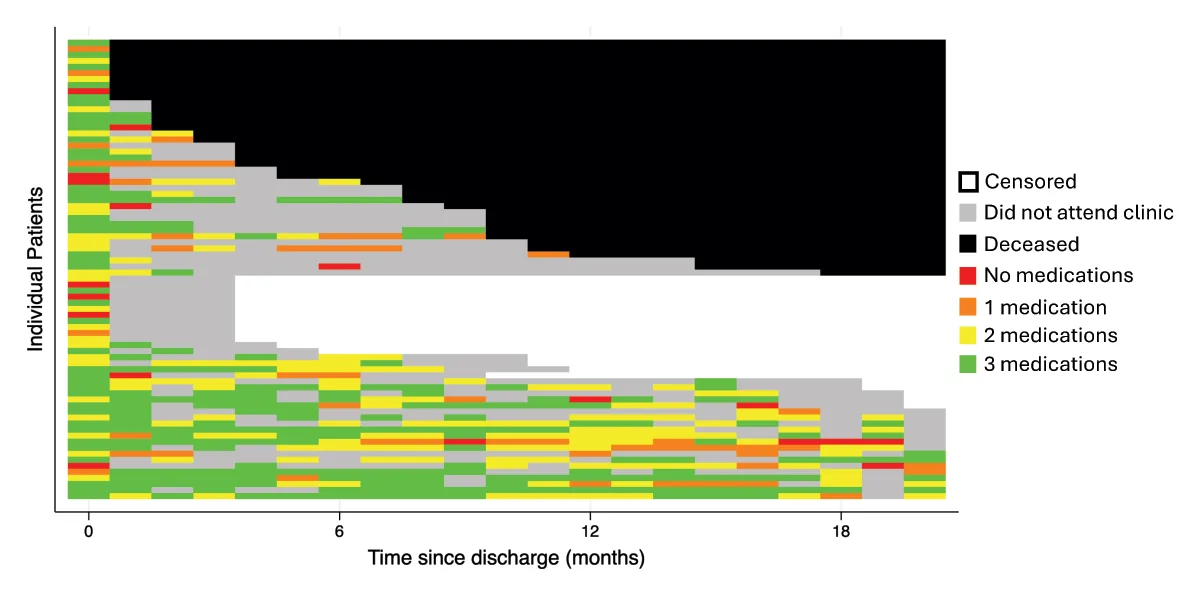
Heatmap of GDMT use for HFrEF patients (n = 76). Heatmap showing utilization of GDMT among patients with HFrEF who survived to hospital discharge; all participants had at least 20 possible months of follow up between discharge and the end of the study period. Each row represents an individual patient and each column one additional month of follow up, with the first column representing hospital discharge. The number of medications indicates the number of heart failure drug classes prescribed (beta-blocker, ACEi/ARB, MRA). For example, “3 medications” indicates all three classes were prescribed. Patients were considered deceased if they were confirmed dead or likely dead during adjudication. Patients were censored after the last known encounter if they missed three consecutive visits.

## Discussion

This retrospective study of patients admitted to BMC with HF in 2023 found high rates of post-discharge mortality and identified gaps in the delivery of guideline-directed care. Importantly, out of the HF care metrics assessed in this study, post-hospital care engagement was associated with a significant increase in survival. This finding was seen for both early linkage-to-care after discharge and retention in care three months after hospitalization. In comparing the outcomes observed in this study to a similar cohort at BMC conducted in 2013 (12), there was no improvement in survival over a 10-year period. These findings highlight that HF outcomes will likely not improve without specific efforts to bolster adoption of national and international HF guidelines, improve linkage to and retention in outpatient care, and facilitate sustained utilization of optimized GDMT.

Consistent with other global heart failure cohorts (5,6) and our own prior work at BMC (12), patients admitted with HF in Northwestern Tanzania were younger, with a median age of 61 years, than is observed in high-income settings. Many of the patients in this study were diagnosed with HF at the time of hospital admission, which is typical in most countries in sub-Saharan Africa, as access to primary care remains limited (13). As expected in populations with HF, comorbidities—co-existing kidney disease (58%), hypertension (47%), diabetes (18%), and atrial arrhythmias (18%)—were common. This highlights both the medical complexity of the HF population and the need for integrated care of multiple chronic diseases. Providers working in resource-limited settings need context-relevant guidelines to help treat patients with HF and multi-morbidity—a key gap in the WHO’s PEN and PEN-plus guidelines (7,8,9).

Patients who present with HF and advanced kidney disease have a significantly increased risk of inpatient mortality (12,14). Among our cohort, we observed high rates of inpatient and early mortality in the first year after hospital discharge, which is consistent with other studies of HF patients in Africa (5,12,14). There were key gaps in the quality of care delivered to HF patients, which had a large impact on post-hospital survival: First, only 79% of patients admitted with HF received appropriate diagnostic testing. While this was not associated with an increased risk of death, it may preclude providers from appropriately phenotyping HF patients or having enough information to safely and confidently initiate or titrate GDMT, which relies on knowledge of patients’ underlying renal function and serum potassium. Secondly, only half of patients with HFrEF were on recommended GDMT at the time of discharge, and prescription of beta-blockers was particularly low. There is a well-described ‘treatment-risk paradox’ in HF, where the most ill patients are also the patients most likely to benefit from GDMT and the least likely to receive it (15,16). Additional research is needed to understand specific barriers to GDMT initiation and continuation in sub-Saharan Africa, where there is often a high rate of concomitant poverty, malnutrition, and comorbidity. While the use of GDMT at the time of discharge was not associated with post-hospital survival, this likely reflects the fact that GDMT at the time of discharge did not guarantee future care engagement and consistent enough outpatient GDMT exposure to derive benefit. In fact, not a single patient in this study received all three classes of GDMT at every time point during the follow-up period.

Notably, only 68% of patients attended their first scheduled post-discharge follow-up appointment, and those who did had a substantial improvement in survival, with a 68% reduction in the adjusted hazard of death. A similar pattern was seen for retention in care at three months after discharge, where those who remained in care for the first three months had a 64% reduction in the adjusted hazard of death. Early linkage to outpatient care and retention in care interventions following diagnosis or hospitalization have been transformational and fundamental to the success of HIV and other chronic disease programs in sub-Saharan Africa (17,18), and transitional care programs are recommended in both European and American HF guidelines (19,20). This study highlights that those efforts must be extended to the HF population in sub-Saharan Africa and coupled with programs to optimize sustained utilization of GDMT—a key barrier to survival in both LMICs and in the United States (21,22,23). As seen in the Figure 5 heat map displaying GDMT use for patients with HFrEF at each month after discharge, sustained care engagement leads to some GDMT exposure, but additional efforts are needed to ensure patients receive appropriate therapy at each clinic visit.

The strength of the association between post-hospital care engagement and survival requires additional confirmation. The analytic approach utilized in this study was designed to mitigate survivor bias by restricting the analysis to patients who survived at least one month or three months after discharge; however, residual survival bias is possible. Additionally, the strength of the association may be limited by residual confounding by factors such as patient frailty, severity of disease, and geographic proximity to follow-up care, all of which may make patients less likely to engage in follow-up care and die after discharge.

Interestingly, in this cohort, insurance status was not associated with inpatient or outpatient mortality and, apart from outpatient beta-blockers, uninsured patients were more likely to be prescribed GDMT. This highlights that financial determinants alone do not dictate outcomes or utilization patterns of low-cost, high-value therapies for patients with HF. Additional work is needed to identify and understand barriers to sustained outpatient care engagement and utilization of GDMT in clinics caring for HF patients and address the financial burden of HF on patients from low-income communities.

Finally, the prescription of GDMT at hospital discharge and in outpatient clinics, as outlined in national and international heart failure guidelines, was low. There is significant evidence from LMICs that access to and utilization of GDMT among HF patients remains low, and mortality is high (5,6). While studies have described implementation strategies for rapid GDMT initiation, like the recent STRONG-HF trial (24), these strategies are not reflective of the patients being treated in many LMICs who are taking little to no medication at the time of HF diagnosis and have minimal prior exposure to outpatient care, as well as limited access to serial laboratory monitoring. Pragmatic research is needed to develop, test, and scale strategies to initiate and sustain long-term GDMT use for patients with HF in resource-limited settings. Early results from ongoing studies to evaluate the efficacy of single-pill combinations or polypills are promising (25,26); however, even these innovative strategies will need to be paired with interventions to identify patients with HF, foster linkage to care and long-term care engagement, and improve facility-level adoption and adherence to national and international guidelines.

There are several important limitations to this study. First, it is a single-center study that may not be generalizable to other settings in Tanzania, East Africa, or other LMICs. However, within Tanzania, BMC is a relatively well-resourced, large academic medical center that offers the highest (or comparable) level of HF care available. Patients receiving care at less well-resourced facilities are unlikely to have better outcomes than those observed at BMC. Secondly, it is possible that some HF patients in this study who were not reachable by phone and out of care at BMC were indeed alive and had transferred care to another center, but this is unlikely: Transfer is often noted in the chart, and patients have many reasons to maintain access to their phone numbers, including their connection to mobile banking. Third, the population of patients captured may not represent the entire population of patients who present to BMC with HF. The fact that the cohort and its outcomes are similar to prior data from BMC, however, supports that these data are likely representative of patients with HF who receive care at BMC (12); furthermore, the data presented here are pragmatic in that they represent the patients most likely to receive inpatient and outpatient care for HF. Fourth, because this is a pragmatic study of the current standard of care, there were missing data for some exposures, including ejection fraction, kidney function, and hemoglobin. While primary analyses assumed normal values for unmeasured parameters, this may have limited our statistical power to identify associations between these exposures and post-hospital outcomes. Nevertheless, our findings remain consistent across both complete-case and worst-case (abnormal) sensitivity analyses. Finally, BMC serves as a referral center for the Lake Zone of Tanzania and, thus, the patients treated at BMC include patients who are referred and patients who present to the emergency department directly. Thus, the patients included in this study may not be representative of the patients who present to smaller hospitals that often serve as the first point of contact for patients with acute medical conditions.

In conclusion, this study describes persistently high rates of inpatient and post-discharge HF mortality and identifies key gaps in the delivery of quality guideline-stipulated HF care. Patients who were linked to and retained in post-discharge care had improved survival rates. Very few patients received consistent access to lifesaving HF medications in the outpatient setting. Evidence-based, context-relevant interventions focused on the diagnosis of HF, linkage to and retention in care, and delivery of quality outpatient care—including optimized GDMT—are needed to improve HF outcomes in Tanzania and other LMICs.

## Additional Files

The additional files for this article can be found as follows:

10.5334/gh.1593.s1 Supplemental Table 1. Factors associated with inpatient and post-hospital mortality.

10.5334/gh.1593.s2 Supplemental Table 2. Sensitivity analyses of factors associated with post-discharge mortality (n = 154).

10.5334/gh.1593.s3 Supplemental Table 3. Sensitivity analyses of factors associated with inpatient and post-hospital mortality, excluding those with no echocardiogram, hemoglobin, or eGFR.

10.5334/gh.1593.s4 Supplemental Table 4. Sensitivity analyses of factors associated with inpatient and post-hospital mortality, assuming those with missing values for echocardiogram, hemoglobin, or eGFR were abnormal.

## Data Availability

The data that support the findings of this study are not publicly available but are available from the corresponding author upon request.
