# Supplementary material for: Heart Failure Care and Post-Hospital Outcomes in Northwestern Tanzania
Source: Glob Heart. 2026 Oct 26;21(1):77. doi: 10.5334/gh.1593 (PMC13722015; doi:10.5334/gh.1593)
Supplement: Supplementary file 1 [file gh-21-1-1593-s1.pdf]

Supplemental Table 1: Factors associated with inpatient and post-hospital mortality.

*ORs derived from univariate and multivariate logistic regression.

**HRs derived from univariate and multivariate Cox proportional hazards regressions.

	Inpatient mortality during index hospitalization (n = 177)*		Post-hospital mortality among those who survived to discharge (n = 154)**	
	Unadjusted OR [95% CI]	Adjusted OR [95% CI]	Unadjusted HR	Adjusted HR [95% CI]
Age (per 10-year increase)	0.88 [0.72, 1.09]	0.81 [0.60, 1.08]	1.11 [0.97, 1.27]	1.13 [0.96, 1.34]
Female [ref: male]	0.37 [0.15, 0.96]	0.27 [0.08, 0.88]	0.90 [0.55, 1.47]	0.99 [0.58, 1.71]
Self-pay [ref: insured]	1.05 [0.43, 2.57]	0.81 [0.22, 3.02]	1.15 [0.70, 1.89]	1.32 [0.73, 2.38]
New heart failure diagnosis [ref: established diagnosis]	0.83 [0.10, 6.96]	0.54 [0.05, 5.51]	2.81 [1.26, 6.27]	2.31 [0.94, 5.70]
HFrEF: EF ≤ 40% [ref: LV EF > 40% or EF unknown]	0.79 [0.33, 1.91]	0.47 [0.16, 1.39]	0.91 [0.56, 1.49]	0.79 [0.45, 1.39]
HTN	0.35 [0.13, 0.94]	0.33 [0.09, 1.17]	1.01 [0.62, 1.65]	0.67 [0.36, 1.27]
Diabetes	0.39 [0.09, 1.77]	0.33 [0.05, 1.99]	1.18 [0.63, 2.21]	1.21 [0.60, 2.47]
Atrial fibrillation or flutter	2.37 [0.88, 6.37]	2.46 [0.62, 9.71]	1.20 [0.66, 2.17]	1.33 [0.68, 2.60]
CKD [ref: CKD Stage 1 and 2—eGFR ≥ 60 or eGFR not measured]	1.00 [1.00, 1.00]			
CKD Stage 3 (eGFR 30 - 59)	1.70 [0.56, 5.21]	3.80 [0.97, 14.81]	1.46 [0.81, 2.65]	1.36 [0.70, 2.65]
CKD Stage 4 (eGFR 15 -29)	2.30 [0.59, 8.96]	4.28 [0.78, 23.48]	1.51 [0.68, 3.34]	1.06 [0.42, 2.72]
CKD Stage 5 (eGFR < 15)	5.23 [1.36, 20.10]	8.62 [1.82, 40.73]	2.73 [1.27, 5.87]	2.56 [1.09, 6.00]
Moderate or severe anemia (Hb < 11.0 mg/dL) [ref: normal hemoglobin, mild anemia, or hemoglobin missing]	1.33 [0.53, 3.37]	2.18 [0.65, 7.33]	1.84 [1.09, 3.08]	1.53 [0.83, 2.84]

\
