# Supplementary material for: Heart Failure Care and Post-Hospital Outcomes in Northwestern Tanzania
Source: Glob Heart. 2026 Oct 26;21(1):77. doi: 10.5334/gh.1593 (PMC13722015; doi:10.5334/gh.1593)
Supplement: Supplementary file 2 [file gh-21-1-1593-s2.pdf]

Supplemental Table 2: Sensitivity analyses of factors associated with post-discharge mortality (n = 154). HRs derived from univariate and multivariate Cox proportional hazards regressions. Participants who could not be reached by phone and were out of care for at least 3 months were handled as follows in each model:

Model 1: Censored at the time of their last contact with the health care system.
Model 2: Vital status adjudicated by the study team.
Model 3: Assumed to be dead.

	**Model 1**		**Model 2**	**Model 3**
	Unadjusted HR	Adjusted HR [95% CI]	Adjusted HR [95% CI]	Adjusted HR [95% CI]
Age (per 10-year increase)	1.11 [0.97, 1.27]	1.13 [0.96, 1.34]	1.11 [0.96, 1.29]	1.06 [0.92, 1.22]
Female [ref: male]	0.90 [0.55, 1.47]	0.99 [0.58, 1.71]	0.88 [0.55, 1.41]	0.77 [0.49, 1.23]
Self-pay [ref: insured]	1.15 [0.70, 1.89]	1.32 [0.73, 2.38]	1.35 [0.80, 2.26]	1.57 [0.94, 2.63]
New heart failure diagnosis [ref: established diagnosis]	2.81 [1.26, 6.27]	2.31 [0.94, 5.70]	2.41 [1.04, 5.59]	2.03 [0.88, 4.68]
HFrEF [ref: LV EF > 40% or EF unknown]	0.91 [0.56, 1.49]	0.79 [0.45, 1.39]	0.82 [0.50, 1.35]	0.83 [0.51, 1.35]
HTN	1.01 [0.62, 1.65]	0.67 [0.36, 1.27]	0.79 [0.45, 1.38]	0.95 [0.55, 1.63]
Diabetes	1.18 [0.63, 2.21]	1.21 [0.60, 2.47]	1.41 [0.75, 2.64]	1.48 [0.80, 2.72]
Atrial fibrillation or flutter	1.20 [0.66, 2.17]	1.33 [0.68, 2.60]	1.10 [0.60, 2.02]	1.19 [0.66, 2.15]
CKD [ref: CKD Stage 1 and 2—eGFR ≥ 60 or eGFR not measured]				
CKD Stage 3 (eGFR 30–59)	1.46 [0.81, 2.65]	1.36 [0.70, 2.65]	1.06 [0.60, 1.87]	1.19 [0.68, 2.10]
CKD Stage 4 (eGFR 15–29)	1.51 [0.68, 3.34]	1.06 [0.42, 2.72]	0.75 [0.32, 1.75]	0.99 [0.45, 2.18]
CKD Stage 5 (eGFR < 15)	2.73 [1.27, 5.87]	2.56 [1.09, 6.00]	1.75 [0.81, 3.82]	1.68 [0.78, 3.65]
Moderate or severe anemia (Hb < 11.0 mg/dL) [ref: normal hemoglobin, mild anemia, or hemoglobin missing]	1.84 [1.09, 3.08]	1.53 [0.83, 2.84]	1.34 [0.77, 2.32]	1.41 [0.83, 2.40]
