# Supplementary material for: Heart Failure Care and Post-Hospital Outcomes in Northwestern Tanzania
Source: Glob Heart. 2026 Oct 26;21(1):77. doi: 10.5334/gh.1593 (PMC13722015; doi:10.5334/gh.1593)
Supplement: Supplementary file 3 [file gh-21-1-1593-s3.pdf]

Supplemental Table 3: Sensitivity analyses of factors associated with inpatient and post-hospital mortality, excluding those with no echocardiogram, hemoglobin, or eGFR.

*Adjusted ORs derived from multivariate logistic regression.
**Adjusted HRs derived from multivariate Cox proportional hazards regressions.

	**Inpatient mortality during index hospitalization (n = 140)***	**Post-hospital mortality among those who survived to discharge (n = 126)****		
		**Model 1**	**Model 2**	**Model 3**
	**Adjusted OR [95% CI]**	Adjusted HR [95% CI]	Adjusted HR [95% CI]	Adjusted HR [95% CI]
Age (per 10-year increase)	0.92 [0.62, 1.37]	1.16 [0.95, 1.42]	1.16 [0.97, 1.38]	1.08 [0.92, 1.28]
Female [ref: male]	0.50 [0.12, 2.00]	0.93 [0.50, 1.71]	0.81 [0.47, 1.37]	0.70 [0.42, 1.17]
Self-pay [ref: insured]	1.12 [0.25, 5.14]	1.41 [0.74, 2.71]	1.49 [0.84, 2.67]	1.79 [1.00, 3.19]
New heart failure diagnosis [ref: established diagnosis]	1.10 [0.09, 13.61]	2.14 [0.66, 6.96]	2.53 [0.88, 7.31]	2.07 [0.73, 5.86]
HFrEF [ref: LV EF > 40%]	1.09 [0.25, 4.72]	0.78 [0.40, 1.52]	0.74 [0.42, 1.32]	0.76 [0.44, 1.32]
HTN	0.49 [0.10, 2.27]	0.70 [0.34, 1.44]	0.77 [0.41, 1.45]	0.97 [0.53, 1.76]
Diabetes	0.63 [0.08, 4.68]	1.33 [0.59, 3.00]	1.46 [0.71, 2.99]	1.54 [0.77, 3.09]
Atrial fibrillation or flutter	4.48 [0.98, 20.47]	1.17 [0.54, 2.54]	1.03 [0.51, 2.07]	1.15 [0.59, 2.26]
CKD [ref: CKD Stage 1 and 2—eGFR ≥ 60]				
CKD Stage 3 (eGFR 30–59)	4.10 [0.72, 23.39]	1.05 [0.52, 2.13]	0.84 [0.46, 1.55]	0.99 [0.54, 1.80]
CKD Stage 4 (eGFR 15–29)	3.68 [0.45, 30.32]	0.69 [0.24, 2.04]	0.55 [0.21, 1.45]	0.82 [0.34, 1.95]
CKD Stage 5 (eGFR < 15)	4.90 [0.59, 41.04]	1.26 [0.44, 3.58]	1.06 [0.41, 2.70]	1.00 [0.40, 2.54]
Moderate or severe anemia (Hb < 11.0 mg/dL) [ref: normal hemoglobin or mild anemia]	2.80 [0.65, 12.00]	1.97 [1.02, 3.77]	1.60 [0.88, 2.90]	1.67 [0.95, 2.94]
