# Supplementary material for: Heart Failure Care and Post-Hospital Outcomes in Northwestern Tanzania
Source: Glob Heart. 2026 Oct 26;21(1):77. doi: 10.5334/gh.1593 (PMC13722015; doi:10.5334/gh.1593)
Supplement: Supplementary file 4 [file gh-21-1-1593-s4.pdf]

Supplemental Table 4: Sensitivity analyses of factors associated with inpatient and post-hospital mortality, assuming those with missing values for echocardiogram, hemoglobin, or eGFR were abnormal.

*Adjusted ORs derived from multivariate logistic regression.
**Adjusted HRs derived from multivariate Cox proportional hazards regressions.

	**Inpatient mortality during index hospitalization (n = 140)***	**Post-hospital mortality among those who survived to discharge (n = 126)****		
		**Model 1**	**Model 2**	**Model 3**
	**Adjusted OR [95% CI]**	Adjusted HR [95% CI]	Adjusted HR [95% CI]	Adjusted HR [95% CI]
Age (per 10-year increase)	0.82 [0.62, 1.09]	1.15 [0.97, 1.36]	1.13 [0.97, 1.31]	1.07 [0.93, 1.24]
Female [ref: male]	0.46 [0.16, 1.35]	1.00 [0.59, 1.72]	0.88 [0.55, 1.41]	0.78 [0.49, 1.23]
Self-pay [ref: insured]	0.79 [0.23, 2.71]	1.34 [0.74, 2.42]	1.36 [0.81, 2.31]	1.61 [0.95, 2.73]
New heart failure diagnosis [ref: established diagnosis]	0.59 [0.06, 5.67]	2.24 [0.89, 5.64]	2.33 [0.99, 5.46]	1.98 [0.85, 4.61]
HFrEF [ref: LV EF > 40%]	1.79 [0.52, 6.13]	0.92 [0.51, 1.66]	0.84 [0.50, 1.40]	0.83 [0.50, 1.36]
HTN	0.58 [0.16, 2.03]	0.74 [0.39, 1.40]	0.79 [0.45, 1.39]	0.95 [0.55, 1.63]
Diabetes	0.31 [0.05, 1.84]	1.30 [0.63, 2.66]	1.50 [0.80, 2.82]	1.57 [0.85, 2.92]
Atrial fibrillation or flutter	2.50 [0.68, 9.10]	1.27 [0.65, 2.47]	1.09 [0.59, 2.00]	1.18 [0.66, 2.13]
CKD [ref: CKD Stage 1 and 2—eGFR $\geq$ 60]				
CKD Stage 3 (eGFR 30–59)	3.91 [0.93, 16.37]	1.20 [0.61, 2.33]	1.00 [0.56, 1.79]	1.12 [0.63, 1.99]
CKD Stage 4 (eGFR 15–29)	5.45 [0.95, 31.23]	0.86 [0.33, 2.25]	0.66 [0.28, 1.58]	0.91 [0.41, 2.02]
CKD Stage 5 (eGFR < 15)	4.44 [1.07, 18.51]	1.40 [0.62, 3.18]	1.25 [0.62, 2.55]	1.24 [0.61, 2.52]
Moderate or severe anemia (Hb < 11.0 mg/dL) [ref: normal hemoglobin or mild anemia]	1.60 [0.56, 4.57]	2.02 [1.15, 3.54]	1.81 [1.10, 2.98]	1.81 [1.12, 2.93]
